# New Insights Into the Role of Qa-2 and HLA-G Non-classical MHC-I Complexes in Malignancy

**DOI:** 10.3389/fimmu.2018.02894

**Published:** 2018-12-06

**Authors:** Istéfani L. da Silva, Lucía Montero-Montero, Enio Ferreira, Miguel Quintanilla

**Affiliations:** ^1^Center of Biological Sciences and Health, Federal University of the West of Bahia, Barreiras, Brazil; ^2^Instituto de Investigaciones Biomédicas Alberto Sols, Consejo Superior de Investigaciones Científicas, Universidad Autónoma de Madrid, Madrid, Spain; ^3^Laboratory of Compared Pathology, Department of General Pathology, Biological Science Institute, Federal University of Minas Gerais, Belo Horizonte, Brazil

**Keywords:** Qa-2, HLA-G, MHC-I, immunosurveillance, cancer

## Abstract

It is well established that the immune system can identify and destroy neoplastic transformed cells in a process known as immunosurveillance. Most studies have focused on the classical major histocompatibility complex (MHC) class Ia molecules, which are known to play an important role on the presentation of tumor antigens to the immune system in order to activate a response against tumor cells. However, a larger family of non-classical MHC class Ib-related molecules has received less attention. In this mini-review, we discuss the role of class Ib murine Qa-2 and its proposed human HLA-G homolog on immunosurveillance during embryogenesis and cancer. Whereas, both HLA-G and Qa-2 are involved in immune tolerance in pregnancy, the current evidence suggests that they play opposite roles in cancer. HLA-G appears to promote tumor progression while Qa-2 acts as a tumor suppressor awaking the immune system to reject tumor cells, as suggested by studies on different cancer cell models, such as melanoma, lymphoma, lung carcinoma, and our own results in mammary carcinoma.

## Introduction

The concept of cancer immunoediting with its three distinct phases: elimination (immunosurveillance), equilibrium (persistence/dormancy) and escape (progression) integrates the capacity of the immune system to both protect the host from cancer and promote cancer development (1). Tumor antigens are presented by the major histocompatibility complex (MHC) class I molecules on antigen-presenting cells (APCs) and recognized by CD8^+^ T cells, which differentiate into cytotoxic T lymphocytes under co-stimulation of CD4^+^ T cells. In addition, tumor cells are targets of innate immune cells, such as macrophages and natural killer (NK) cells. Thus, neoplastic cells can be recognized and destroyed by both the innate and adaptive immune systems. But if antitumor immunity is unable to completely eliminate them, tumor variants may survive and enter into the equilibrium phase, where cells and cytokines of adaptive immunity restrain tumor outgrowth. These dormant tumor cells may eventually escape the control of immune cells and progress to clinically detectable malignancies. The immune system contributes to tumor progression by selecting more aggressive tumor variants and allowing cancer cells to survive and growth in immunocompetent hosts. Tumor cells that escape the control of immune cells secrete factors that inhibit effector immune cell function and recruit inflammatory and regulatory immune cells that generate an immunosuppressive microenvironment and promote cancer progression (2).

## The Human HLA-G Complex in Physiology and Cancer

The human leukocyte antigen G (HLA-G) belongs to the non-classical MHC complex, or class Ib, that has a potent immunomodulatory activity in pathophysiological situations requiring immune tolerance, such as fetus tolerance during pregnancy, autoimmune and inflammatory diseases, and acceptance of allograft transplantation in patients (3). The diversity of HLA-G products occur by alternative splicing of a primary transcript, which gives rise to four membrane-bound (G1-G4) and three soluble (G5-G7) protein isoforms (4). In addition, soluble isoforms can be generated by proteolytic cleavage of membrane-bound HLA-G forms. While the basic structure of HLA-G is similar to that of classical HLA class Ia molecules, they differ in that HLA-G is less polymorphic (5). HLA-G modulates the innate and adaptive immune systems by interacting with inhibitory receptors on the surface of immune cells, such as the immunoglobulin-like transcript 2 (ILT2) and ILT4 on dendritic cells, ILT2 and the killer immunoglobulin-like receptor 2DL4 (KIR2DL4) on NK cells, and ILT2 on T cells and monocyte/macrophages (4, 6, 7). By binding to these receptors, HLA-G directly inhibits immune cells. However, it has been reported that upon binding to KIR2DL4 HLA-G activates NK cells and promotes cytotoxicity and IFN-γ secretion (8, 9). Furthermore, other studies did not find evidence for a functional interaction between HLA-G and KIR2DL4 in NK cells (10). Therefore, whether HLA-G is a ligand of KIR2DL4 is at present a matter of controversy (11). In addition, several immune cells, including T cells, APCs and a subset of dendritic cells can express or secrete HLA-G. Thus, APCs expressing HLA-G1 induces CD4^+^ T cell differentiation into regulatory T suppressor cells that block cytotoxic T lymphocyte function (12). These Treg cells are important to sustain immune tolerance and prevent autoimmune diseases. In normal tissues, the most abundant expression of HLA-G is on the surface of trophoblasts in the placenta where can effectively suppress the local immune response in the uterus and promote maternal tolerance to the fetus (13). Apart from this specific function, HLA-G shares with classical HLA class Ia molecules the ability to present antigens (14, 15), suggesting a role for HLA-G on the immune defense against infection and anti-cancer response.

**Figure 1:**
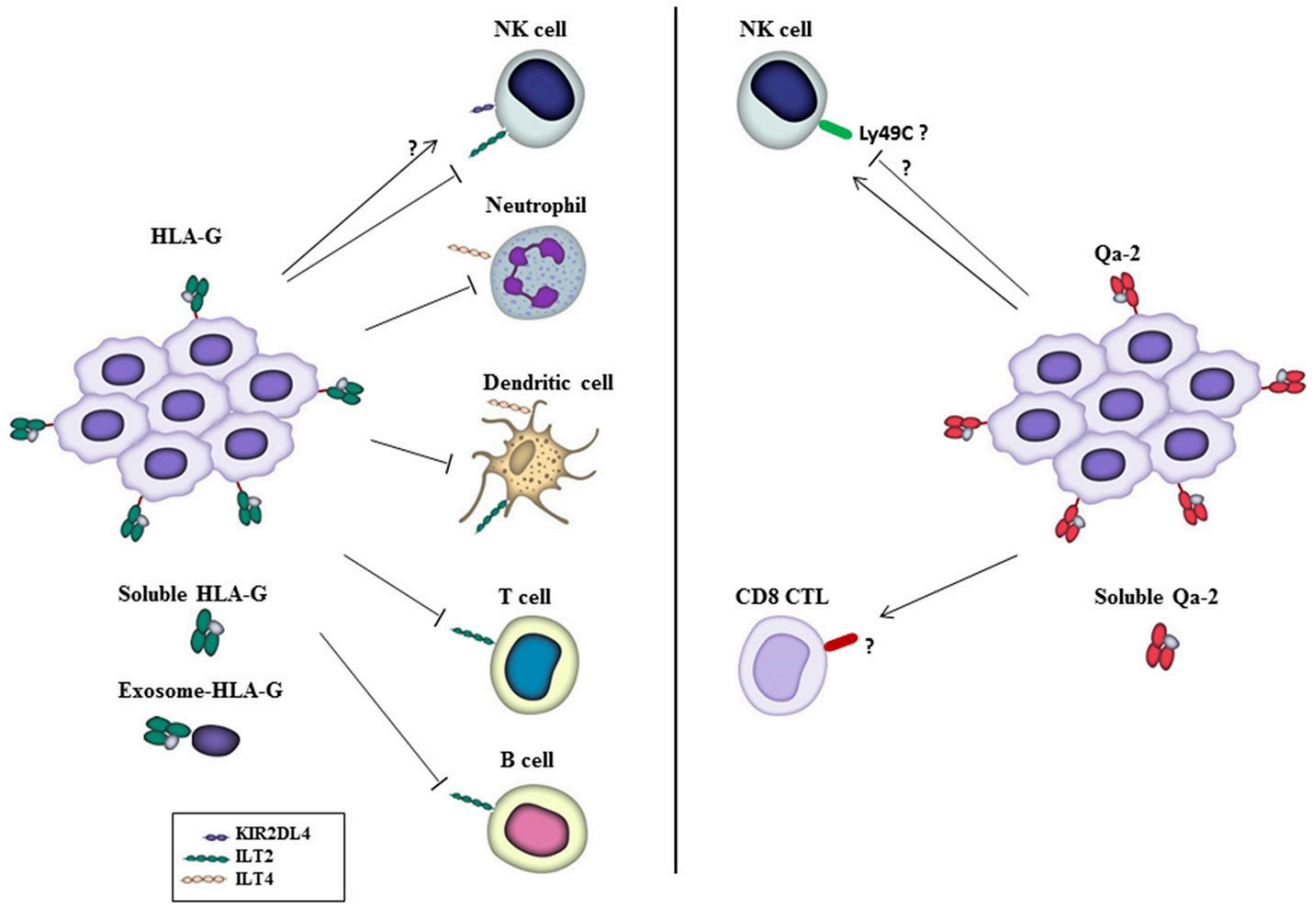
Functions of HLA-G and Qa-2 on immune cells in the tumor microenvironment. HLA-G, either on the surface of tumor cells or in its soluble form or associated with exosomes, inhibits different traits of immune cells through interaction with the inhibitory receptors ILT2 and ILT4, allowing tumor cells to escape from immune surveillance. Also, HLA-G has been reported to interact with KIR2DL4 receptor to activate NK cells, although this is a matter of controversy (see the text). On the other hand, Qa-2 has been reported to activate NK cells and CD8^+^ CTLs that inhibit tumor cell growth and mediate tumor rejection. Inhibition of NK cells by Qa-2 was also reported *in vitro*, and Ly49C postulated as a Qa-2 inhibitory receptor, see the text. Receptors on immune cells involved in the response to Qa-2 are presently unknown. Likewise, whether Qa-2 is released to the extracellular milieu associated to exosomes remains to be investigated.

However, while downregulation of classical HLA class Ia molecules is common to most cancers, overexpression of membrane-bound and soluble HLA-G proteins have been found in tumors, including lymphomas, leukemias, melanoma, and breast, kidney, ovarian, lung, esophageal, gastric, pancreatic and colorectal carcinomas. The majority of studies find HLA-G expression in solid tumors to be associated with malignancy and poor prognosis (3, 4, 16–18). In fact, HLA-G is considered as a target for cancer gene therapy (19). However, there are some reports showing the opposite. Thus, for example, upregulation of HLA-G levels was found a favorable prognosis factor in triple-negative breast carcinomas (20). In contrast to solid tumors, HLA-G levels did not show any clear correlation with patient outcome in hematological malignancies (4). HLA-G can modify the action of innate immune cells by inducing tolerance in APCs and inhibiting NK-cell mediated killing (see Figure 1). Thus, APCs expressing HLA-G in the tumor microenvironment have suppressive properties and are partly responsible for tumor immune escape. On the other hand, soluble HLA-G secreted by both tumor and immune cells may directly inhibit CD4^+^ and CD8^+^ T cell proliferation (21). In fact, plasma levels of soluble HLA-G are higher in patients with pancreatic cancer compared to healthy donors, and soluble HLA-G levels inversely correlate with the number of peripheral activated T cells (22). It is worth to mention that soluble HLA-G levels are significantly higher in benign lesions than in malignant tumors and can be used as a diagnosis tool to distinguish premalignant from malignant stages (6, 23). HLA-G molecules can be transferred from tumor cells to activated NK cells and other immune cells by a process called trogocytosis, which involves the delivery of plasma membrane fragments from one cell to another leading to downregulation of the immune response (7, 24). This constitutes an effective mechanism for tumor cells to evade surveillance of surrounding immune cells. On the other hand, HLA-G has been found to be secreted associated to extracellular vesicles; i.e., exosomes originated by the endo-lysosomal pathway, derived from melanoma cells (25) and placental trophoblasts (26), which provide another mechanism for tumors to modulate the host immune response and for trophoblasts to modify the maternal immunological environment.

## The Murine Non-Classical MHC Complex Qa-2. Its Role in Embryogenesis and Autoimmunity

Qa-2 has been reported to be the HLA-G homolog in mice (27). Both HLA-G and Qa-2 molecules have membrane-bound and soluble forms that originate by alternative splicing, display immunoregulatory roles, and are involved in embryonic development (7). There are four major Qa-2 loci: *Q6, Q7, Q8*, and *Q9*, which localize at the Qa region of chromosome 17 and are present in different combinations in each mouse haplotype (28). Since *Q6* and *Q8* loci are almost perfect duplicate of each other, and *Q7* and *Q9* only differ by a single nucleotide leading to a change of Gln in *Q7* to Glu in *Q9*, they are referred as *Q6*/*Q8* and *Q7*/*Q9* pairs. Therefore, Qa-2 gene clusters seem to have evolved by sequential duplication of a primordial gene pair consisting of one “odd” locus that originated *Q7* and *Q9* and one “even” locus that gave rise to *Q6* and *Q8* (27). The pair *Q6/Q8* encodes a transmembrane protein while the pair *Q7/Q9* encodes a glycosylphosphatidylinositol (GPI)-anchored membrane protein. The GPI linkage facilitates clustering of Qa-2 protein on lipid rafts and suggests a potential role for Qa-2 in cell signaling (29). *Q7/Q9* may generate a soluble Qa-2 isoform by alternative splicing, in which loss of exon 5 results in the inability of the product to attach the GPI anchor (30, 31). In addition, alike to HLA-G, soluble Qa-2 isoforms can also be generated by shedding of membrane-bound forms (32).

Qa-2 expression on the cell surface requires the assembly within the endoplasmic reticulum of a trimer. Qa-2 associates with β2 microglobulin and is loaded with a small peptide, which is primarily produced in the cytosol by the ubiquitin–proteasome pathway and then transported into the lumen of the endoplasmic reticulum by the transporter associated with antigen processing (TAP) protein, a heterodimer consisting of TAP1 and TAP2 subunits (33). Qa-2 binds a wide array of TAP-dependent self and non-self-nonameric peptides, which suggests Qa-2 has an immunosurveillance function alerting for the presence of intracellular pathogen infections or neoplastic transformation (34). Thus, Qa-2 restricted CD8^+^ T cells contribute to protect the host against infection with *Mycobacterium tuberculosis* (35) and polyoma virus (36), the latter requiring CD4^+^ T cells for its maintenance phase (37). Likewise, CD8^+^ T cells are involved in the anti-tumor immune response elicited by Qa-2 (see below).

Qa-2 genes were identified as Ped (preimplantation embryo development) candidate genes that modulate the rate of embryo cleavage division and subsequent fetal survival (38, 39). Embryos from some mouse strains develop faster than those of other strains, depending on the presence of Qa-2 protein (i.e., the products of *Q7*/*Q9* pair) on the surface of preimplantation embryo. This has been named the *Ped fast* phenotype (39). It was proposed that Qa-2 protein protects the developing fetus from the attack of maternal NK cells and/or macrophages, which could account for the higher rate of fetal loss observed in embryos lacking Qa-2 protein (40). In addition, besides an overall reproductive advantage, the presence of Qa-2 protein in the embryo grants a healthier life to the adult mouse (41).

In adult tissues, Qa-2 is widely distributed but generally at low levels. It is present in APCs, immature and mature thymocytes, thymic epithelial cells, intestinal epithelial cells and immunologically privileged sites/cells, such as the anterior chamber of the eye, hair follicles and sperm in testis (31, 32, 42). Qa-2 has been involved in T lymphocyte proliferation, particularly in CD4^+^ T cells (43). However, the α3 domain of Qa-2 is unable to interact with CD8 in order to effectively activate cytotoxic T cells (44). Qa-2 has been reported to mediate signal transduction pathways, as antibody-induced crosslinking of Qa-2 promoted proliferation of resting T cells depending on Fyn (a member of the Src family of kinases), PI-3 kinase and Akt (45). Also, Qa-2 is required for the selection of intraepithelial unconventional CD8αα/TCRαβ T cells, which appear to be regulatory cells involved in maintaining intestinal integrity (46).

HLA-G allelic variants inducing a significantly lower expression level of HLA-G products are genetic risk factors for Behcet's disease, a chronic multi-systemic disorder involving gastrointestinal, mucocutaneous, ocular, vascular, central nervous and articular systems (47). A similar role for Qa-2 was also shown in a Behcet's disease-like mouse model induced by the herpex virus simplex, in which silencing of Qa-2 by intravenous injection of specific siRNA worsened the disease symptoms (48).

Acute graft rejection is the main complication in organ transplantation. It involves allograft recognition by the immune system as non-self and graft destruction by reactive immune cells (49). It has been reported that patients with upregulated HLA-G expression in grafts and plasma have better allograft acceptance that HLA-G-negative patients (50, 51). Lu and coworkers have studied the relationship of Qa-2 expression in grafts and peripheral blood lymphocytes (PBLs) with graft rejection in a well-established murine skin transplantation model in the presence or absence of immunosuppressive agents. They found negative or weakly positive Qa-2 expression in mice without immunosuppressive treatment similar to that of control mice. In contrast, treatment with immunosuppressive drugs upregulated Qa-2 expression in both grafts and PBLs and prolonged the survival of skin allografts. Qa-2 expression subsequently decreased during allograft rejection (52). These results clearly point to an inverse relationship between Qa-2 levels and allograft rejection.

Whereas, HLA-G is a well-established ligand of the ILT family of immune inhibitory receptors (4, 6, 7), little is known about the receptors for Qa-2 in immune cells. A potential candidate is paired immunoglobulin-like inhibitory receptor (PIR-B), the murine ortholog of human ILT, which has been shown to interact with MHC class I molecules (53) and HLA-G (54, 55). However, to our knowledge, evidence for a functional interaction between PIR-B and Qa-2 is still lacking. In a recent review, Goodall and co-workers claim that Qa-2 binds Ly49C on NK cells (56). Ly49C is a member of the lectin-like Ly49 family receptors, the functional counterparts in mice of human KIR receptors. However, the experimental demonstration for this interaction has not been published yet.

## The Role of Qa-2 In Cancer

Similarly to classical HLA class Ia molecules, Qa-2 expression was found to be downregulated in tumors and derived cell lines, including melanoma, hepatoma, lymphoma, leukemia, and mastocytoma, which suggested to the authors a suppressive role for Qa-2 in cancer (42). In accordance with this hypothesis, Chiang and Stroynowski demonstrated that restoration of *Q9* expression in melanoma, lung carcinoma and T-cell lymphoma cell lines that have downregulated Qa-2 expression resulted in a CD8^+^ cytotoxic T lymphocyte (CTL)-mediated immune response that inhibited *in vivo* tumor growth in syngeneic hosts (57–59).

The lack of Qa-2 expression in B16 and B16-derived melanoma cell lines with low and high metastatic capacities suggest that downregulation of Qa-2 levels is an early event during melanoma progression. Experiments with genetically manipulated melanoma cells showed that TAP-2-dependent restoration of *Q9* expression led to reduced tumorigenic potential in syngeneic mice, and *in vivo* depletion studies indicated that both NK cells and CD8^+^ CTLs collaborated in the rejection response (57) (Figure 1). It should be mentioned, however, that *Q9* was previously shown to partially protect melanoma cells from lysis by activated NK cells *in vitro*, suggesting that *Q9* may interact with an inhibitory NK receptor (60) that might correspond to Ly49C (56). Nevertheless, *in vivo* studies indicate that *Q9* sensitizes tumors to trigger a NK cell-mediated cytolytic response either by directly killing tumor cells or by stimulating downstream T cell responses through secretion of cytokines and chemokines. Interestingly, the protection conferred by *Q9* to the host was stronger that the anti-tumor effect exerted by the expression of its structurally homologous class Ia H2-K^b^ antigen (57). Further studies demonstrated that *Q9* protein is a target for anti-tumor immune surveillance, as CD8^+^ T-cell-deficient mice as well as β2 microglobulin and CD8-knockout mice were unable to reject *Q9*-bearing melanoma tumors. Moreover, the display of peptide-loaded *Q9* on the surface of melanoma cells induced strong immunological memory (58). The ability of *Q9* to act as a restriction element for anti-tumor CTL is not specific of melanoma cells, as *Q9* surface expression on 3LLA9F1 Lewis lung carcinoma or RMA T cell lymphoma cell lines also induces a potent anti-tumor CTL response that eliminate tumor cells in syngeneic hosts. Mice challenged with one *Q9*-expressing tumor develop immunological memory against subsequent challenge with different *Q9*-bearing tumor cells, suggesting that *Q9* has the capacity to present tumor antigens shared by distinct lineages of cancer cells (59). Like *Q9, Q8* protein is ubiquitously distributed in mouse normal tissues and is frequently downregulated in tumors (42). Despite *Q8* differs significantly from *Q9* in the amino acid sequence of α1 and α2 domains involved in peptide binding and T cell recognition, *Q8* and *Q9* have overlapping binding motifs and exhibit cross-reactive CTL responses recognizing shared tumor-associated antigens from melanoma, Lewis lung carcinoma and T cell lymphoma (61). Whereas, the studies of Chiang and Stroynowski suggest that Qa-2 activates innate and adaptive immune cells to reject tumors, they hold some limitations that should be taken into account. First, these studies come from only one laboratory and should be confirmed by others. Second, the B78H1melanoma cell model used in these experiments is not completely negative for MHC class I molecules, but express low levels of HLA class I antigens (http://www.lollini.it/b78.htm), and the possibility that they were upregulated after TAP-2 transfection was not ruled out experimentally.

We have addressed the role of Qa-2 in tumor growth and progression using the 4T1 mammary carcinoma cell model. 4T1 tumor cells are representative of the highly aggressive triple-negative subtype of human breast cancer (62). In the 4T1 cell line, only a small percentage of cells (about 4%) express Qa-2 on the cell surface, and Qa-2 protein levels were further reduced during *in vivo* tumor growth and in tumor-derived cultured cells (63), suggesting that Qa-2 expression is downregulated during breast cancer development. Cell lines derived from tumors induced by 4T1 in the back skin, or in the mammary fat pad, of syngeneic Balb/c mice elicited a partial epithelial-mesenchymal transition (EMT) and exhibited increased stem cell characteristics and enhanced tumor-initiating and invasive capacities that correlated with reduced Qa-2 expression. In fact, Qa-2 expression was completely lost in a CD44^high^/CD24^med/low^ cancer stem cell subpopulation (64) isolated from these cell lines (63). This striking result suggests the possibility that Qa-2 is excluded from cancer stem cells, thus contributing to their evasion from immunosurveillance. Nonetheless, this hypothesis deserves further investigation. Downregulation of Qa-2 in cancer stem cells appears to be mediated, at least partially, by the Src signaling pathway. Indeed, pharmacological inhibition of Src kinase activity enhanced Qa-2 expression and concomitantly reduced stemness of 4T1 tumor-derived cell lines (63). Increased Src activity is a general characteristic of malignancy associated with EMT, stemness, invasion and metastasis (65). In order to confirm the anti-tumor role of Qa-2, we forced the expression of *Q7* in 4T1 cells. *Q7* is a key member of the Qa-2 family in Balb/c mice (42). 4T1 cells overexpressing *Q7* produced tumors that grew slower and were less metastatic than control or parental cells (63).

Interestingly, Servín-Blanco and colleagues have reported that Qa-2-derived peptides elicited an anti-tumor immune response against carcinomas induced by 4T1 cells in Balb/c mice resulting in a significant inhibition of tumor growth and a reduction in the number of metastatic lesions (66). This possibility of immunotherapy targeting Qa-2 is shared by HLA-G, as HLA-G-derived peptides were able to induce a CTL response against HLA-G-expressing human renal carcinoma cells (67).

## Concluding Remarks

In summary, Qa-2 has been found to have a different role in cancer with respect to its putative human homolog HLA-G. Whereas, HLA-G seems to help tumor cells to escape from immunosurveillance by directly interacting with inhibitory receptors that halt innate and adaptive immune cells, Qa-2 appears to activate NK and CD8^+^ CTLs to reject tumors. Thus, despite similarities in structure and function during embryogenesis and autoimmunity, both families of HLA class lb proteins act in an opposite manner in the context of tumors (Table 1). Therefore, downregulation of Qa-2 and upregulation of HLA-G expression are major immune evasive mechanisms used by murine and human tumors, respectively. Nevertheless, in contrast to HLA-G, only a handful of reports have addressed the functional implication of Qa-2 in tumors, and mainly using a limited number of cell lines. Thus, studies on the role of Qa-2 in cancer should be extended. Specially, *in vivo* models of carcinogenesis with normal or genetically engineered mice should be used in order to ascertain more accurately the mechanisms of tumor suppression exhibited by these molecules. Moreover, it would be of great interest to clarify the apparent inverse relationship between Qa-2 and cancer stem cells using both *in vitro* and *in vivo* models of cancer. Particularly relevant to understand the different roles of HLA-G and Qa-2 in cancer is to identify the specific receptors for Qa-2 in different immune cells, either inhibitory or stimulatory, and the regulatory signals triggered upon its binding.

**Table 1:** Functional characteristics of HLA-G and Qa-2.

	**HLA-G**	**Qa-2**
Immune tolerance during pregnancy Autoimmune disease Graft rejection Tumor growth	Promotes Prevents Protects Promotes	Promotes Prevents Protects Inhibits

## Author Contributions

IdS and MQ wrote the manuscript. LM-M and EF reviewed it.

### Conflict of Interest Statement

The authors declare that the research was conducted in the absence of any commercial or financial relationships that could be construed as a potential conflict of interest.
